# Associations of Atherogenic Lipid Ratios and Visceral Adiposity Indices with Cardiovascular Risk Scores in Rheumatoid Arthritis

**DOI:** 10.5152/ArchRheumatol.2026.26338

**Published:** 2026-05-11

**Authors:** Uğur Güngör Demir, Ali Nail Demir, Alper Uysal

**Affiliations:** 1 Physical Medicine and Rehabilitation Clinic, Mersin City Training and Research Hospital, Mersin, Türkiye; 2 Rheumatology Clinic, Mersin City Training and Research Hospital, Mersin, Türkiye

**Keywords:** Atherogenic lipid ratios, cardiovascular risk, Framingham Risk Score, rheumatoid arthritis, SCORE, visceral adiposity indices

## Abstract

**Background/Aims:**

Rheumatoid arthritis (RA) is associated with increased cardiovascular risk beyond traditional risk factors. Recently, atherogenic lipid ratios and visceral adiposity indices have emerged as potential markers of cardiometabolic risk in chronic inflammatory diseases. This study aimed to examine their associations with estimated cardiovascular risk scores in patients with RA.

**Materials and Methods:**

This cross-sectional study included 198 patients with RA. Individuals with established cardiovascular disease or major comorbid conditions were excluded. Atherogenic lipid ratios, including the Atherogenic Index of Plasma (AIP), Atherogenic Coefficient (AC), Castelli Risk Index (CRI)-1, CRI-2, and triglycerides (TG)/high-density lipoprotein cholesterol (HDL-C) ratio, were calculated. Visceral adiposity indices included the Visceral Adiposity Index (VAI), Lipid Accumulation Product (LAP), and Waist-Triglyceride Index. Ten-year cardiovascular risk was estimated using the modified Framingham Risk Score (FRS) and the modified Systematic Coronary Risk Evaluation (SCORE) system.

**Results:**

Atherogenic lipid ratios, particularly AC, CRI-1, and CRI-2, demonstrated weak but statistically significant positive correlations with both modified SCORE and modified FRS. In contrast, adiposity-related indices (LAP and VAI) and atherogenic lipid ratios (AIP and the TG/HDL-C ratio) were significantly associated with the modified FRS but not with the modified SCORE. No significant associations were observed between RA disease activity measures and cardiovascular risk scores.

**Conclusion:**

In RA, cardiovascular risk is linked closely to adiposity and lipid disturbances than current disease activity. Modified FRS may better capture adiposity-related cardiovascular risk than modified SCORE, and incorporation of atherogenic lipid and visceral adiposity indices may improve risk stratification.

**Main Points**

- Atherogenic lipid ratios and visceral adiposity indices were associated with estimated cardiovascular risk in rheumatoid arthritis (RA) patients, whereas disease activity measures were not.
- Modified Framingham Risk Score (FRS) showed stronger and more consistent associations with adiposity-related and atherogenic indices than modified SCORE.
- Indices reflecting visceral adiposity (Lipid Accumulation Product, Visceral Adiposity Index, and Waist-Triglyceride Index) were associated with modified FRS but not with modified SCORE.
- These findings support the potential value of incorporating atherogenic lipid ratios and visceral adiposity markers into cardiovascular risk assessment, particularly when using FRS-based models in RA.

## Introduction

As a systemic autoimmune condition, rheumatoid arthritis (RA) is distinguished by chronic synovitis, leading to cartilage erosion and deformation of the joints.^1^ Rheumatoid arthritis also presents with systemic involvement, and cardiovascular comorbidities are among its most common extra-articular manifestations.^2^

Of these cardiovascular comorbidities, accelerated atherosclerosis exerts the greatest clinical impact because of its close relationship with cardiovascular and cerebrovascular disorders.^3^ Despite significant progress in elucidating the pathogenesis and treating RA, patients’ life expectancy remains below that of the general population.^4^^,^^5^ A recent study indicates that the standardized mortality ratio for fatal myocardial infarction in RA ranges from 0.99 to 3.82, while for fatal stroke, it ranges from 1.08 to 2.00.^6^ Another study reported that cardiovascular mortality in individuals with RA is approximately 48% greater than that observed in the general population. This increase is particularly pronounced in deaths due to myocardial infarction and reaches up to 68%.^7^ Such an excessive atherosclerotic burden cannot be entirely explained by conventional cardiovascular risk factors.^8^ There is a large body of evidence about the close relationship between dyslipidemia and cardiovascular risk.^9^ Dyslipidemia appears to manifest in RA patients and some medication may contribute to this atherogenic lipid phenotype.^10^^-^^12^ The variable burden of chronic inflammation in RA leads to differences in lipid concentrations across disease stages, complicating the interpretation of their impact on cardiovascular risk.^13^ A number of novel indices are available for assessing cardiovascular risk. The Atherogenic Index of Plasma (AIP) represents the equilibrium between cardioprotective and pro-atherogenic lipoproteins and is closely related to lipoprotein particle size. Compared to the untransformed variable, the AIP better satisfies the normality assumption of the residuals in the analysis conducted to assess overall treatment effects.^14^ Compared with conventional lipid measures, AIP is less affected by changes in RA disease activity.^13^ Atherogenic Coefficient (AC) underscores the importance of high-density lipoprotein cholesterol (HDL-C) in the evaluation of cardiovascular risk.^15^ As composite lipid ratios, Castelli Risk Index (CRI-1) and CRI-2 provide greater predictive value for vascular risk than individual lipid parameters.^16^ Moreover, patients with RA exhibit increased adipose tissue mass, which may be characterized by greater inflammation and metabolic dysfunction compared with matched non-RA controls.^17^ Visceral Adiposity Index (VAI), Lipid Accumulation Product (LAP), and Waist-Triglyceride Index (WTI) have emerged as novel indices for identifying visceral obesity and predicting cardiovascular and cerebrovascular events.^18^^-^^22^ This study examines how the previously mentioned indicators might be linked to cardiovascular disease risk in patients diagnosed with RA.

## Methods

The study enrolled 198 rheumatoid arthritis patients who met the 2010 American College of Rheumatology/European League Against Rheumatism classification criteria.^23^ A history of established cardiovascular disease such as heart failure, coronary artery disease, peripheral artery disease, symptomatic carotid artery disease, and abdominal aortic aneurysm was an exclusion criterion. Other exclusion criteria were pregnancy, malignancy, active infection, renal failure, liver disease, obstructive sleep apnea, and chronic obstructive pulmonary disease.

The study received ethical clearance from Ethics Committee of Mersin City Training and Research Hospital (approval date: December 24, 2025; decision number 2025/66). As this was a retrospective study, participants were not asked to provide written informed consent. Three established scoring systems including the Disease Activity Score in 28 joints (DAS28), the Clinical Disease Activity Index (CDAI), and the Simplified Disease Activity Index (SDAI) were employed to quantify disease activity levels.^24^ After fasting overnight for 12 hours, blood specimens were drawn and tested for various inflammatory markers, including C-reactive protein (CRP) and erythrocyte sedimentation rate (ESR), and metabolic parameters such as low-density lipoprotein cholesterol (LDL-C), HDL-C, triglycerides (TG), total cholesterol (TC), and fasting blood glucose.

Anthropometric measurements were collected from all participants. Weight, height, body mass index (BMI), and waist circumference (WC) were recorded while participants wore light clothing and no shoes. Waist circumference was measured midway between the lower margin of the last rib and the upper boundary of the iliac crest following a relaxed exhalation.^25^

Lipid Accumulation Product is a practical and cost-effective index of central lipid accumulation. The LAP was computed separately based on sex, using the formula (WC − 58) × TG for females and (WC − 65) × TG for males.^26^ Visceral Adiposity Index was estimated using sex-specific formulas. In females: VAI = [WC/(36.58 + 1.89 × BMI)] × (TG/0.81) × (1.52/HDL-C); in males: VAI = [WC/(39.68 + 1.88 × BMI)] × (TG/1.03) × (1.31/HDL-C).^^18^^ Waist-Triglyceride Index is an anthropometric index calculated by multiplying WC by TG.^27^

Atherogenic Index of Plasma was computed as log_10_ of the TG to HDL-C ratio.^28^ The AC, CRI-1, CRI-2, and TG/HDL-C were calculated using standard formulas (TC − HDL-C)/HDL-C for AC; TC/HDL-C for CRI-1; and LDL-C/HDL-C for CRI-2.^15^^,^^29^

Metabolic syndrome (MetS) was defined according to the updated harmonized International Diabetes Federation criteria. It was diagnosed when at least 3 cardiometabolic components were present: central obesity, elevated TG, reduced HDL-C, elevated blood pressure, and impaired fasting glucose.^30^

Ten-year cardiovascular disease risk was estimated using modified Framingham Risk Score (FRS) and modified Systematic Coronary Risk Evaluation (SCORE).^31^^,^^32^ European League Against Rheumatism advises increasing cardiovascular risk estimates by 50% in patients with RA to reflect their elevated risk burden.^33^^,^^34^

The modified FRS, which incorporates variables such as sex, age, blood pressure, smoking status, and cholesterol levels, categorizes individuals into 3 cardiovascular risk levels: low (less than 10% risk over 10 years), intermediate (10%-20%), and high (greater than 20%). According to the SCORE algorithm, cardiovascular risk was stratified into 4 categories: low (<1%), moderate (1%-5%), high (5%-10%), and very high (greater than 10% or presence of established cardiovascular disease).^31^^-^^33^

### Statistical Analysis

Statistical analyses were performed via Statistical Package for Social Sciences (SPSS) for Windows, version 21.0 (IBM SPSS Corp.; Armonk, NY, USA). Visual and analytical methods were used to evaluate whether the variables followed a normal distribution. Continuous data are presented as mean ± SD and categorical data are reported as numbers and percentages. Nonparametric comparisons of continuous variables were performed with the Mann–Whitney *U* and Kruskal–Wallis tests. Spearman’s correlation coefficient was used to evaluate relationships between numerical data. Correlation coefficients were classified as poor (<0.30), fair (0.30-0.59), or strong (≥0.60) based on commonly used medical thresholds.^35^^,^^36^

## Results

Overall, 198 patients with RA were enrolled. The mean age was 54.59 ± 11.48 years, and 143 patients (72.2%) were female. The mean disease duration was 8.62 ± 7.30 years. Demographic characteristics, clinical features, laboratory findings, and treatment profiles of the patients are presented in Table 1.

Correlation analyses between metabolic indices and cardiovascular risk scores are summarized in Table 2. The modified SCORE showed significant positive correlations with the AC (*r* = 0.170, *P* = .017), CRI-1 (*r* = 0.170, *P* = .017), and CRI-2 (*r* = 0.200, *P* = .006). There were no significant correlations between modified SCORE and LAP, VAI, AIP, TG/HDL-C ratio, or WTI (all *P* > .05). The modified FRS demonstrated significant positive correlations with LAP (*r* = 0.214, *P* = .002), VAI (*r* = 0.148, *P* = .038), AIP (*r* = 0.214, *P* = .002), AC (*r* = 0.294, *P* < .001), CRI-1 (*r* = 0.294, *P* < .001), CRI-2 (*r* = 0.300, *P* < .001), WTI (*r* = 0.145, *P* = .042), and TG/HDL-C ratio (*r* = 0.214, *P* = .002). There were no significant correlations between disease activity measures (DAS28, CDAI, and SDAI) and either modified SCORE or modified FRS (all *P* > .05).

## Discussion

This study showed the associations between atherogenic lipid ratios, visceral adiposity indices, and estimated cardiovascular risk scores in RA. The present study reported that atherogenic lipid ratios, particularly AC and CRI-1/CRI-2, were consistently but weakly associated with both modified SCORE and modified FRS. Whereas some adiposity-related indices (LAP and VAI) and some atherogenic lipid ratios (AIP and the TG/HDL-C) were associated with modified FRS but not with modified SCORE. No statistically significant association was identified between disease activity indices and cardiovascular risk scores. This finding shows that cardiovascular risk in RA may be more closely related to adiposity-related indices and atherogenic lipid ratios than to current disease activity.

However, many treatments used in RA, such as glucocorticoids, methotrexate, tumor necrosis factor (TNF) inhibitors, and Janus kinase (JAK) inhibitors, can affect lipid levels and atherogenic indices. For this reason, these treatments may act as important confounding factors when evaluating lipid- and adiposity-related cardiovascular markers in cross-sectional studies.^11^^,^^13^^,^^17^^,^^37^ Recent studies show that RA treatments, especially JAK inhibitors like upadacitinib, increase TC levels and affect vascular stiffness more than TNF inhibitor therapy.^37^ These treatment-related metabolic changes may affect the interpretation of lipid- and adiposity-related cardiovascular risk markers in RA.

Previous studies show that traditional cardiovascular risk prediction models may fail to fully capture the actual risk in chronic inflammatory diseases. This underestimation may be due to the “lipid paradox” in RA, in which lower LDL-C and TC levels are linked to elevated cardiovascular risk as a result of inflammation.^^17^^ Recent evidence also shows that lipid ratios like TC/HDL-C and composite lipoprotein measures are independently associated with cardiovascular risk in RA, which suggests that inflammation-related lipid changes may limit the accuracy of conventional risk scores.^34^ Dessein et al^^38^^ found that FRS-based scores better discriminate subclinical atherosclerosis and cardiovascular risk compared with SCORE in RA patients. Omma et al^^39^^ showed that FRS was significantly associated with visceral adiposity–related parameters, particularly the VAI, but not with SCORE in patients with psoriatic disease. This difference may be due to how the 2 models define outcomes. SCORE focuses only on fatal cardiovascular events,^32^ while the FRS considers both fatal and non-fatal events.^40^ This may explain why FRS-based scores were more strongly associated with adiposity-related and atherogenic indices in the cohort. These observations are consistent with the results, which show that adiposity-related indices and atherogenic lipid ratios were more closely associated with the modified FRS than with modified SCORE. Because smoking is heavily weighted in the SCORE model,^32^ the presence of smoking in the cohort may have reduced the association between SCORE and visceral adiposity–related indices. Visceral fat is now considered an important contributor to cardiometabolic risk in inflammatory arthritis. Studies show that BMI alone may not accurately capture inflammation-related fat distribution or mortality risk in RA, while composite obesity indices better reflect visceral adiposity and associated cardiometabolic risk.^41^ Lipid Accumulation Product, VAI, and WTI were associated with modified FRS but not with modified SCORE in the study*.*

Omma et al^^39^^ reported that no significant association was observed between psoriatic disease activity scores and either the Framingham Score or SCORE. They suggested that the cross-sectional structure of their study and the subjects’ medication use could alter the results of this relationship. Their results support the finding that existing disease activity measures may not accurately represent cardiovascular risk in RA. Solomon et al^^42^^ showed that RA patients who had lower average disease activity over time had a lower risk of cardiovascular events. However, no relationship was found between disease activity measures and cardiovascular risk scores. Although this long-term study shows that sustained control of RA disease activity lowers cardiovascular events, cross-sectional studies using standard cardiovascular risk scores may not reflect this relationship. A cross-sectional study found weak but statistically significant correlations between the FRS and disease activity indices like DAS28 and SDAI in active RA patients.^43^ However, a similar association was not observed in the cohort. This difference may be due to the fact that most of the patients had non-severe disease activity under treatment. Risk scores like FRS rely largely on age and traditional risk factors and do not include inflammatory burden.^31^^,^^33^ Therefore, in patients with well-controlled RA, disease activity may have little influence on estimated cardiovascular risk.

Atherogenic Coefficient, CRI-1, and CRI-2 showed significant correlations with both modified FRS and modified SCORE, whereas AIP and TG/HDL-C were associated only with modified FRS in the study. Many studies suggest that atherogenic lipid ratios are useful for evaluating cardiovascular risk in RA. In a previous study, atherogenic lipid ratios, such as CRI-1, CRI-2, AC, TG/HDL-C ratio, and AIP, were found to be elevated in RA patients compared with healthy subjects. Systemic inflammation in RA may change lipid levels, making single lipid measurements less reliable for assessing cardiovascular risk and favoring the use of composite lipid indices.^29^ Bonilla et al^^44^^ found that Castelli risk indices showed more reliable links with cardiovascular risk than standard lipid levels in individuals with periodontitis, including those with RA. This result suggests that combined lipid ratios may better represent atherogenic changes in chronic inflammation.

The findings suggest that cardiovascular risk in RA is more strongly influenced by adipose- and lipid-related changes than by current disease activity levels. While systemic inflammation is known to worsen lipid profiles and atherogenic indices in RA,^29^^,^^33^ the lack of a direct relationship between disease activity scores and cardiovascular risk in the study suggests that long-term metabolic factors may be more important.

Although this study was not designed to explore causal mechanisms, several pathophysiological factors may help explain the findings. Chronic systemic inflammation in RA may lead to qualitative changes in lipid particles by affecting lipoprotein composition and function. These changes may not be fully reflected by absolute lipid concentrations but are better captured by atherogenic lipid ratios.^29^ Bag-Ozbek and Giles describe that HDL-C can transform into pro-inflammatory HDL-C (piHDL-C) in patients with RA. This transformation results from cytokine-induced changes in its protein and enzymatic composition, leading to a loss of its antioxidative and anti-atherogenic properties.^^17^^

Additionally, metabolic dysfunction related to visceral adiposity contributes to insulin resistance, dyslipidemia, and proatherogenic states in inflammatory arthritis, increasing cardiovascular risk independently of current disease activity.^39^^,^^45^ These processes may affect risk models that include metabolic factors, such as FRS-based scores, more than SCORE-based models that mainly rely on traditional risk factors.^39^

The findings indicate that traditional cardiovascular risk scores differ in their ability to reflect cardiometabolic disturbances associated with RA. Especially, FRS-based scores showed significant correlations with visceral adiposity and atherogenic lipid indices, including LAP, VAI, AIP, and TG/HDL-C ratio, whereas modified SCORE failed to detect such associations. In the cohort, MetS was present in 45.5% of patients, which is higher than the 32% prevalence reported in a recent meta-analysis of RA populations.^46^ Another study by Loganathan et al^^47^^ reported a pooled prevalence of MetS of 31% in RA populations. This discrepancy may arise from differences in study populations, diagnostic definitions, treatment patterns, or the use of metabolically sensitive risk models like the modified FRS.

Overall, the results and the literature suggest that cardiovascular risk assessment in this population may benefit from incorporating atherogenic lipid ratios and adiposity markers, particularly when FRS-based models are used instead of SCORE-based tools.^29^^,^^38^^,^^39^^,^^41^^,^^45^

From a clinical perspective, these findings highlight the potential utility of incorporating atherogenic lipid ratios and visceral adiposity indices into routine cardiovascular risk assessment in patients with RA. Given that traditional risk scores may underestimate cardiovascular risk in this population, particularly when relying primarily on conventional lipid parameters or disease activity measures, the inclusion of composite lipid indices (such as AC, CRI-1, and CRI-2) and adiposity-related markers (such as LAP and VAI) may provide a more comprehensive evaluation of cardiometabolic risk. This approach may be especially relevant in patients with well-controlled disease activity, where residual cardiovascular risk persists despite apparent inflammatory control.

This study has several limitations. First, because of the cross-sectional design, causal relationships between atherogenic lipid ratios, visceral adiposity indices, and estimated cardiovascular risk scores cannot be determined. Prospective studies are needed to clarify how metabolic alterations relate to cardiovascular risk over time in patients with RA. Second, cardiovascular risk was evaluated using prediction models rather than actual cardiovascular events. Despite their widespread use, modified SCORE and to a lesser extent modified FRS may still underestimate cardiovascular risk in inflammatory diseases such as RA. Accordingly, the results should be viewed in the context of predicted, not actual, cardiovascular events. Third, disease activity was assessed at a single time point. As a result, cumulative inflammation over time and its effects on lipid metabolism and long-term cardiovascular risk may be underestimated. Similarly, information on cumulative inflammatory exposure adjusted for disease duration was also lacking. Fourth, while multiple lipid ratios and visceral adiposity indices were examined, newer cardiovascular risk biomarkers, including advanced lipoprotein subfractions and inflammatory markers, were not assessed. Finally, the results may not be generalizable to all RA populations due to the single-center design.

In conclusion, this study demonstrates that atherogenic lipid ratios (CRI-1, CRI-2, AC, AIP, and TG/HDL-C) and indices of visceral adiposity (VAI, LAP, and WTI) are associated with estimated cardiovascular risk in patients with RA, whereas disease activity measures are not consistently linked to cardiovascular risk scores. Some atherogenic lipid ratios (CRI-1, CRI-2, and AC) were weakly but consistently associated with both modified SCORE and modified FRS, whereas indices of visceral adiposity (VAI, LAP, and WTI) were associated with modified FRS but not with modified SCORE.

These findings suggest that assessing cardiovascular risk in RA may be improved by incorporating atherogenic lipid ratios and adiposity-related indices into traditional risk scores, particularly when using FRS-based models. Although causality cannot be established, the results indicate that adiposity- and lipid-related alterations may have a greater impact on estimated cardiovascular risk in this population than current disease activity. These findings contribute to the growing evidence that cardiometabolic alterations rather than current inflammatory disease activity play a central role in cardiovascular risk stratification in RA. This study supports a more integrated and metabolically informed approach to risk assessment in this population.

### Data Availability Statement:

The data that support the findings of this study are available on request from the corresponding author.

### Artificial Intelligence Usage Statement:

The authors declared that no Artificial Intelligence Tool was used in the preparation of the manuscript.

### Ethics Committee Approval:

This study was approved by the Ethics Committee of Mersin City Training and Research Hospital (Approval No: 2025/66, Date: December 24, 2025).

### Informed Consent:

N/A.

### Peer-review:

Externally peer-reviewed.

### Author Contributions:

Concept – U.G.D., A.N.D.; Design – U.G.D., A.U.; Supervision – U.G.D., A.N.D., A.U.; Resources – A.N.D, A.U.; Materials – U.G.D., A.U.; Data Collection and/or Processing – U.G.D., A.N.D., A.U.; Analysis and/or Interpretation – U.G.D., A.N.D.; Literature Search – A.N.D, A.U.; Writing – U.G.D., A.U.; Critical Review – U.G.D., A.U., A.N.D.

### Declaration of Interests:

The authors have no conflicts of interest to declare.

### Funding:

The authors declare that this study received no financial support.

## Figures and Tables

**Table 1.** Demographic Data and Clinical Features of Patients

**Features**	**Mean ± SD/n (%)**
Age (mean ± SD)	54.59 ± 11.48
Female/male	143 (72.2) / 55 (27.8)
Disease duration	8.58 ± 7.25
Height (cm)	162.63 ± 9.80
Weight (kg)	75.97 ± 12.48
BMI (kg/m^2^)	29.23 ± 5.16
Tobacco user	42 (21.2)
LAP Index	63.65 ± 42.91
VAI	2.07 ± 1.29
AIP	0.35 ± 0.23
AC	2.64 ± 1.02
CRI-1	3.73 ± 1.01
CRI-2	2.13 ± 0.88
TG/HDL-C ratio	2.67 ± 1.42
Diabetes mellitus, n (%)	39 (15.2)
Dyslipidemia, n (%)	18 (9.1)
Hypertension, n (%)	73 (36.9)
MetS	90 (45.5)
Rheumatoid factor positivity, n (%)	126 (63.64)
Anti-CCP antibodies positivity, n (%)	128 (64.6)
ESR (mean ± SD)	20.16 ± 14.87
CRP (mean ± SD)	9.72 ± 12.91
DAS28 (mean ± SD)	3.25 ± 1.13
SDAI (mean ± SD)	12.69 ± 8.50
CDAI (mean ± SD)	10.84 ± 7.93
Medical therapy, n (%)	​
GCs	129 (65.2)
Methotrexate	112 (56.6)
Leflunomide	34 (17.2)
Sulfasalazine	35 (17.7)
Hydroxychloroquine	136 (68.7)
Rituximab	2 (1)
Tocilizumab	1 (0.5)
Anti-TNF-alpha	14 (7.1)
Tofacitinib	15 (7.6)
Abatacept	3 (1.5)
NSAID	74 (37.4)

AC, Atherogenic Coefficient; AIP, Atherogenic Index of Plasma; Anti-CCP, anti–cyclic citrullinated peptide; Anti-TNF-alpha, anti–tumor necrosis factor alpha; BMI, body mass index; CDAI, Clinical Disease Activity Index; CRI-1, Castelli’s Risk Index I; CRI-2, Castelli’s Risk Index II; CRP, C-reactive protein; DAS28, Disease Activity Score in 28 joints; ESR, erythrocyte sedimentation rate; GCs, glucocorticoids; HDL-C, high-density lipoprotein cholesterol; LAP Index, lipid accumulation product index; MetS, metabolic syndrome; NSAID, nonsteroidal anti-inflammatory drug; SDAI, Simplified Disease Activity Index; TG, triglycerides; VAI, Visceral Adiposity Index.

**Table 2.** Correlations Between Atherogenic and Visceral Adiposity Indices and Cardiovascular Risk Scores in Patients With Rheumatoid Arthritis

​	**Modified SCORE**		**Modified FRS**	
	***r***	***P***	***r***	***P***
LAP Index	0.087	.225	0.214	.002
VAI	0.014	.843	0.148	.038
AIP	0.066	.353	0.214	0.002
AC	0.166	.028	0.283	.009
CRI-1	0.170	.017	0.294	.000
CRI-2	0.200	.006	0.300	.000
TG/HDL-C ratio	0.066	.353	0.214	.002
WTI	0.028	.692	0.145	.042
DAS28	-0.061	.392	0.016	.825
CDAI	-0.111	.118	-0.050	.480
SDAI	-0.049	.490	0.000	.996

AC, Atherogenic Coefficient; AIP, Atherogenic Index of Plasma; CDAI, Clinical Disease Activity Index; CRI-1, Castelli’s Risk Index I; CRI-2, Castelli’s Risk Index II; DAS28, Disease Activity Score in 28 Joints; P Index, Plasma Index; SDAI, Simplified Disease Activity Index; TG/HDL-C ratio, triglyceride to high-density lipoprotein cholesterol ratio; VAI, Visceral Adiposity Index; WTI, Waist-to-Triglyceride Index.
